# Language athletes: Dual-language code-switchers exhibit inhibitory control advantages

**DOI:** 10.3389/fpsyg.2023.1150159

**Published:** 2023-03-31

**Authors:** Leah Gosselin, Laura Sabourin

**Affiliations:** ERP-Ling Laboratory, Department of Linguistics, University of Ottawa, Ottawa, ON, Canada

**Keywords:** code-switching, executive function, inhibitory control, bilingualism, interactional context, adaptive control hypothesis

## Abstract

Recent studies have begun to examine bilingual cognition from more nuanced, experienced-based perspectives. The present study adds to this body of work by investigating the potential impact of code-switching on bilinguals’ inhibitory control abilities. Crucially, our bilingual participants originated from a predominantly dual-language environment, the interactional context which is believed to require (and therefore, potentially train) cognitive control processes related to goal-monitoring and inhibition. As such, 266 French Canadian bilinguals completed an online experiment wherein they were asked to complete a domain-general (Flanker) and a language-specific (bilingual Stroop) inhibitory control task, as well as extensive demographic and language background questionnaires. Stepwise multiple regressions (including various potential demographic and linguistic predictors) were conducted on the participants’ Flanker and Stroop effects. The results indicated that the bilinguals’ propensity to code-switch consistently yielded significant positive (but unidirectional) inhibitory control effects: dual-language bilinguals who reported more habitual French-to-English switching exhibited better goal-monitoring and inhibition abilities. For the language-specific task, the analysis also revealed that frequent *unintentional* code-switching may mitigate these inhibition skills. As such, the findings demonstrate that dual-language code-switchers may experience inhibitory control benefits, but only when their switching is self-reportedly deliberate. We conclude that the bilinguals’ interactional context is thus of primary importance, as the dual-language context is more conducive to intentional code-switching. Overall, the current study highlights the importance of considering individualistic language experience when it comes to examining potential bilingual executive functioning advantages.

## 1. Introduction

Since the turn of the millennium, many studies have reported that individuals who speak multiple languages may benefit from increased executive functioning abilities compared to monolinguals. This Bilingual Advantage Hypothesis (BAH) stems from the idea that bilinguals and multilinguals are constantly managing co-activated languages: according to requirements of a given context, bilinguals must learn to activate their goal-relevant language, and to inhibit their non-pertinent language. This sort of activation–inhibition “tango” is by-and-large considered to be cognitively effortful as it directly implicates inhibitory control processes, thus training the neural substrates which underlie these mechanisms. Indeed, convincing results reveal that bilingualism bolsters positive cognitive effects across the lifespan (see Bialystok et al., 2012 for a review). However, this perspective is still highly controversial. Many studies yield no evidence to support bilingualism-related cognitive benefits; others suggest that positive results are the consequence of unsound methods, and are disproportionately available due to publication biases (see Hilchey and Klein, 2011; Paap and Greenberg, 2013). When bilingual advantages do present themselves, researchers often question whether they are restricted to the modality of language (i.e., “Bilinguals are good language users.”), or whether these benefits extend to cognition more broadly (i.e., “Bilinguals experience general improved executive functioning.”; e.g., Branzi et al., 2016; Tao et al., 2021).

As such, recent reviews push for a less dichotomous view of the topic. Rather than treating bi- or monolingualism as categorical classifiers, there is a call to examine the effects of bilingualism in more nuanced ways (Bak, 2016: 712; see also Woumans and Duyck, 2015). The idea is that some bilinguals may experience executive functioning advantages, but most likely not *all* bilinguals do. This is not particularly problematic, as bilinguals (and monolinguals, for that matter) are not monolithic (De Bruin, 2019; Beatty-Martínez and Titone, 2021; Kałamała et al., 2022).

The question thus stands: What do *advantaged* bilinguals have in common in terms of their language usage? What is it about bilingualism that may trigger certain types of cognitive benefits? To this effect, the present study considers the role of code-switching frequency and language context in bilingual executive functioning. We conducted a within-group study on French-English bilinguals, in order to examine whether their language-specific and domain-general inhibitory control capabilities were predicted by their code-switching habits. Importantly, these adults originated from a primarily dual-language context (i.e., Québec-Ontario regions of Canada).

### 1.1. Inhibitory control in a nutshell

In modern society, individuals must be able to adapt their behaviors, concentrate on goal-relevant information, remember important data, and ignore attractive lures. These paramount mental processes, known as executive functions (EF), are primarily regulated by the prefrontal cortex (e.g., Duncan and Owen, 2000; Otero and Barker, 2014), a region of the brain which is organizationally unique to primates (e.g., Schoenemann et al., 2005).

One core EF is inhibitory control. Inhibitory control (and its subcomponents, conflict and goal monitoring) refers to an individual’s ability to concentrate on important information and ignore potential attention-drawing but irrelevant cues (Diamond, 2013). For instance, a person with high inhibitory control abilities may be very good at rejecting intrusive thoughts during test-taking, avoiding impulsive actions, delaying gratification, and/or ignoring loud or salient distractions. Lower inhibitory control has been linked to poorer mental health (e.g., Tavares et al., 2007), reduced job success (Bailey, 2007) and increased criminal involvement (e.g., Denson et al., 2011).

Both linguistic tasks (e.g., Stroop) and non-linguistic tasks (e.g., Flanker, Simon, and Anti-saccade) are widely used to quantify inhibitory control. Though these paradigms are usually compared across studies, it is important to note that even tasks bearing the same name vary considerably in terms of their design and procedure (e.g., Salo et al., 2001). Nevertheless, these paradigms standardly compare incongruent and congruent trials to neutral trials to operationalize an individual’s inhibitory control abilities. Incongruent trials involve high conflict, wherein a target is presented with antagonistic information (e.g., a left-facing target arrow is flanked by right-facing arrows; the word RED is presented in green font). In contrast, congruent trials involve high conformity, as the target is presented alongside harmonious information (e.g., a left-facing target arrow is flanked by left-facing arrows; the word RED is presented in red font). Finally, neutral trials serve as a baseline: the target therein is presented with neither conflicting nor concordant information (e.g., a left-facing target arrow is flanked by simple dashes; the word HOUSE is presented in green font).

Within the last few decades, researchers have established that inhibitory control is impacted by a diverse range of external factors, including age (Allain et al., 2005), education (Dorbath et al., 2013), fitness level (Guiney and Machado, 2013) and emotional affect (Yang et al., 2013). Crucially, numerous studies have also demonstrated that executive functioning is remarkably trainable (Draganski et al., 2004; Bialystok, 2006; Bialystok and DePape, 2009), with language-related variables like bilingualism sometimes outweighing all other modulating factors (Hartanto and Yang, 2019).

### 1.2. The role of code-switching in inhibitory control

Code-switching (i.e., the alternation of multiple languages within a single communicative event) is a systematic linguistic phenomenon (see Poplack, 1980); it is widespread in multiple stable bilingual communities, including the Francophone population in Canada (Poplack, 1989). Code-switching may occur both inter-sententially (between sentence or clause boundaries) or intra-sententially (within sentence or clause boundaries). French-English code-switching is colloquially known as *Frenglish* or *franglais* in the Canadian context, especially when it involves intra-sentential alternations.

Early research criticized this linguistic behavior, stating that code-switching reflected the speaker’s lack of competence or “linguistic laziness” (see Heredia and Altarriba, 2001). Fortunately, the bulk of linguistic research from the past several decades demonstrates that code-switching is highly rule-governed and systematic (e.g., Di Sciullo et al., 1986; Belazi et al., 1994; MacSwan, 2000; Adamou and Shen, 2019). Furthermore, adults who do code-switch are generally found to be highly proficient in both of their languages (Poplack, 1980). Recent evidence thus clearly illustrates that code-switching is a high-level skill that can be honed through practice and specific in-the-field bilingual language experiences.

If the BAH stems from the idea that bilinguals train their EFs when they rouse and suppress their co-activated languages, researchers’ recent interest in code-switching is quite natural. In theory, code-switchers are performing the activation–inhibition tango even more often than bilinguals who do not code-switch. Since this phenomenon requires inhibitory control, code-switching may thus result in the strengthening of the neural substrates which underlie the executive functioning system. Consider the potential bilingual profiles in (1).

(1) **Potential scenarios of bilingualism:**
  - Réjeanne lives and works in Toronto. Her working day is spent entirely in English. At home, Réjeanne and her family converse in French.
  - Marthe lives and works in Ottawa. Though she typically serves customers in English, she discusses in French with her co-workers.
  - Mona lives and works in Sudbury. She typically greets patients in both languages, and they reply similarly. Mona understands that most patients are also bilingual.

In (1a), Réjeanne performs the activation–inhibition shift between her languages very infrequently: she simply needs to switch from French-mode to English-mode when she goes to work and do the opposite when she returns home. Alternatively, in (1b), Marthe must be quite attentive in monitoring her linguistic environment; she is activating and inhibiting her languages in turn much more regularly. Marthe is also presumably producing inter-sentential code-switches. As such, Marthe is more likely to experience improved inhibitory control abilities since she switches between her languages (i.e., she puts her executive functioning system into practice) more frequently than Réjeanne. These scenarios demonstrate that an individual’s experience with code-switching may play a role in producing bilingual advantages.

A collection of recent empirical research put these conjectures to the test. That is, studies have begun to investigate whether frequent or habitual code-switching is linked to increased executive functioning abilities by using a variety of inhibitory control tasks. Some studies do indeed observe that habitual code-switchers experience inhibitory advantages (Hofweber et al., 2016, 2019, 2020a; Verreyt et al., 2016; Ooi et al., 2018; Hartanto and Yang, 2020; Jylkkä et al., 2020; Sanchez-Azanza et al., 2020; Kheder and Kaan, 2021; Carter et al., 2022; see also López-Penadés et al., 2020 for global reaction time advantages). Interestingly, fMRI research also indicates that frequent code-switchers display higher caudate volumes (Korenar et al., 2022) and that the brain regions involved in language switching, including the caudate (e.g., the left inferior frontal gyrus, anterior cingulate cortex) overlap with the regions normally associated to executive functioning (Abutalebi and Green, 2008; Garbin et al., 2010; Bialystok et al., 2012; Luk et al., 2012; De Baene et al., 2015; Rossi et al., 2021). This suggests that language switching and executive functioning processes implicate the same mechanisms, and further, that practicing one might result in better performance in the other (Bialystok et al., 2012).

Nonetheless, other experiments yield no effect for code-switching frequency (Soveri et al., 2011; Jylkkä et al., 2017, 2018; Paap et al., 2017, 2019; Chan et al., 2020; Kałamała et al., 2020), or even negative switching effects (i.e., bilinguals who code-switch *less* have better executive functioning: Jylkkä et al., 2018, 2021; Hofweber et al., 2020b). The variability in the results is not inherently worrisome: these studies utilized various tasks (e.g., Stroop, Flanker, Go/no-go, Simon, Anti-saccade, Stop-signal, ANT, Elevator task…), tested different participant samples (young, middle-aged, and older adults) in various contexts (e.g., L1-dominant speakers immersed in an L2 environment, simultaneous, early or late bilinguals in a L1 single-language society, multilingual/high-switching environments), and measured code-switching habits in distinct ways (e.g., questionnaires, Likert scales, email analyses, judgment tasks, Ecological Momentary Assessment).

Instead of being problematic, the fluctuations in the literature may allow us to observe patterns about code-switching and inhibitory control. While much evidence supports the idea that code-switchers are unknowingly training their executive functioning system by frequently activating and suppressing their languages (e.g., Hofweber et al., 2020a), it is likely not the case that those who frequently code-switch *automatically* have improved cognitive control skills; many code-switching bilinguals do not outperform non-code-switchers (e.g., Hofweber et al., 2020b). Thus, the present study examines whether the participants’ bilingual interactional context may help explain the complexity of the literature.

### 1.3. Inhibitory control and the bilingual interactional context

The concept of the bilingual interactional context is extremely pertinent to the BAH controversy, given the Adaptive Control Hypothesis (Green and Abutalebi, 2013). This hypothesis postulates that bilinguals exist within distinct interactional contexts, and importantly, that each context imposes differential cognitive control demands on the bilingual speaker. These contexts are largely related to the degree separation between the languages in the environment. In a single-language context, speakers are expected to converse in a single language within a given environment. In a dual-language context, bilinguals may converse in more than one language within a given environment, but usually maintain a single language for different conversations, speakers, or topics. Inter-sentential code-switches may occur, though they are likely contextual. Finally, in a dense code-switching context, bilinguals are free to use multiple languages with the same co-interlocutor and within the same conversation. These three bilingual contexts roughly translate to scenarios (1a), (1b) and (1c), respectively (see section 1.2).

According to the Adaptive Control Hypothesis, the dual-language context is expected to engage an individual’s cognitive control processes, but the dense code-switching context is not. In a dual-language context, individuals must monitor their environment and select one of their co-activated but competing languages (Green and Abutalebi, 2013). They must manage a “gate-like” mechanism which allows back-and-forth suppression of their non-relevant language (Green and Wei, 2014). Contrastively, in a dense code-switching context, speakers expect their fellow interlocutors to be bilingual as well; they seldom need to attend to their environment to select the appropriate language for mutual understanding. Instead, individuals who exist in a dense code-switching context may partake in opportunistic language usage (Green and Abutalebi, 2013). They are able to retrieve lexical items based on ease of access (e.g., frequency, semantic network of the item; Gollan and Ferreira, 2009; Xu et al., 2021), regardless of their language origin. Such an open control mode (Green and Wei, 2014) implies that a bilingual’s languages are functioning cooperatively, rather than competitively. Thus, even though dense code-switching bilinguals are likely the most frequent code-switchers, their switching is not hypothesized to train the substrates underlying cognitive control.

Crucially, the current literature has scarcely disentangled participants’ code-switching frequency from their bilingual interactional context of origin. This means that inconsistencies in the findings may be linked to the confound of the interactional context of the participants in each study. For instance, research yielding null results may have been testing participants who typically exist in dense code-switching contexts; such bilinguals are not expected to possess executive functioning advantages. Some authors do indeed broach the potential impact of language context when discussing their null results, noting that their language background questionnaire did not allow them to disambiguate differences in bilingual contexts (Soveri et al., 2011; Jylkkä et al., 2017; Chan et al., 2020). Other results support the Adaptive Control Hypothesis by showing that it was the dense code-switching that was linked to weaker cognitive control mechanisms (Hofweber et al., 2020a; though see Jylkkä et al., 2018; Kałamała et al., 2020).

A recent trail-blazing experiment corroborated the Adaptive Control Hypothesis (Beatty-Martínez et al., 2021; see also Freeman et al., 2022; van den Berg et al., 2022). In this study, Spanish-English bilinguals from Puerto Rico listened to code-switched and unilingual sentences while the size of their pupil was measured. The idea behind this measure is that an individual’s pupil size is believed to increase in relation to their cognitive effort; situations necessitating higher attentional control are typically accompanied by pupil dilation (Zekveld et al., 2018). Beatty-Martínez and Titone (2021) were able to show that bilinguals who reported typically using their languages in separate contexts (single-, dual-language contexts) displayed pupil dilation in response to code-switches. By contrast, bilinguals who reported using their languages cooperatively (dense code-switching context) did not exhibit pupil dilation when they heard code-switches (for comparable results within experimentally-induced interactional contexts see Han et al., 2022). Production studies have also demonstrated that mandatory code-switching (as in dual-language contexts) is cued by slower response times compared to voluntary code-switching (as in dense code-switching contexts; e.g., Jevtović et al., 2020; Zhu et al., 2022); this suggests that the former is effortful, and may thus train the mechanisms that underlie cognitive control. Nevertheless, some authors have failed to find evidence supporting the Adaptive Control Hypothesis (e.g., Paap et al., 2021).

In brief, qualifying an individual as a “habitual code-switcher” does not necessarily disambiguate their interactional context of origin. While switching can occur in both dual-language and dense code-switching contexts, habitual code-switching is perhaps only advantageous if it occurs deliberately (dual-language context) rather than opportunistically (dense code-switching context). This is because deliberate code-switching occurs when a bilingual monitors their environment, activates their target language, and suppresses their non-relevant language; unintentional code-switching does not require these processes. Returning to our scenarios in (1), we can hypothesize that Mona (1c) is less likely to display a bilingual advantage than Marthe (1b), and possibly even Réjeanne (1a). Our study is thus aimed at examining code-switching among bilinguals who correspond to the profile in (1b).

## 2. The present study

The controversy of the BAH still saturates the field of psycholinguistics. Given the presence of mixed results, we stray away from the monolithic representation of bilingualism, and instead investigate specific bilingual language experiences that may result in cognitive benefits. In particular, the current study considers the role of an individual’s code-switching habits in relation to their inhibitory control abilities. In the past, research examining code-switching has not often disentangled switching frequency and bilingual interactional context of origin. The potential nuance between these two variables demonstrates that, when it comes to examining the BAH, simply comparing bilinguals to monolinguals is insufficient. In fact, even collapsing code-switchers into a monolithic group might introduce confounds.

As such, we test French-English bilinguals who originate from a dual-language context, the environment which is believed to train cognitive control processes (see Abutalebi and Green, 2008; Green and Wei, 2014). If deliberate code-switching does indeed lead to bilingual advantages, this population is presumably the most likely to display benefits. These participants completed a series of online language background questionnaires, which were examined in relation to their performance on two online inhibitory control tasks: a linguistic Stroop task and a non-linguistic Flanker task.

Altogether, our study was guided by the following research questions: Does habitual code-switching within a dual-language context train bilinguals’ inhibitory control abilities? If so, are these advantages restricted to the language-specific domain (i.e., Stroop), or do they extend to domain-general functioning (i.e., Flanker)? We anticipated that more habitual code-switchers would exhibit inhibitory control advantages (i.e., faster reaction times (RTs) for trials requiring goal maintenance and/or conflict monitoring), akin to the bulk of similar past research (Hofweber et al., 2016, 2019, 2020a; Verreyt et al., 2016; Ooi et al., 2018; Hartanto and Yang, 2020; Jylkkä et al., 2020; Sanchez-Azanza et al., 2020; Kheder and Kaan, 2021). Given that neuroanatomical research suggests that language switching can train the general substrates underlying executive functioning (Abutalebi and Green, 2008; Garbin et al., 2010; Bialystok et al., 2012; Luk et al., 2012; De Baene et al., 2015), we expected to observe these inhibitory control advantages for both language-specific and domain-general abilities. Altogether, we hypothesize that dual-language code-switchers may be considered language athletes: instead of strengthening their muscles through physical exercise, they are unknowingly training their executive functioning system by deliberately activating and suppressing their languages on a regular basis.

## 3. Methods

### 3.1. Participants

This study received full ethics approval from the University of Ottawa’s Research Ethics Board (file #S-01-21-6,539). Participants were recruited from two sources. Firstly, recruitment occurred through the Integrated System of Participant Research at the University of Ottawa, where students can partake in research for course credit. Secondly, recruitment scripts were posted to social media pages dedicated to francophones in Ontario (participants recruited through this stream received no compensation). In both cases, the study advertisements specified the following eligibility criteria: participants had to be highly proficient adult speakers of both French and English, have normal or corrected-to-normal vision, and must not have been diagnosed with attention-related disorders or suffered any significant head trauma.

In total, 266 individuals gave informed consent and completed the online study. Forty-one participants were subsequently excluded: 15 self-reported having less than advanced proficiency in French or English, twenty indicated being exposed to a language other than French or English from birth, four had advanced proficiency in a third language, and two were color-blind. The final participant sample thus included 225 individuals (*M_age_* = 21.2 years, *range* = 16–50; 190 women, 35 men; 91.1% right-handed). Refuters of the BAH have previously criticized the small-to-medium sample sizes and lacking statistical power of studies reporting positive results (e.g., Paap et al., 2016), noting that advantages tend to disappear among larger samples (e.g., Paap et al., 2015). As such, an effort was made to test a large sample of bilinguals. A survey of known studies using similar data analysis procedures (see section 3.3.4) revealed that bilinguals’ propensity to produce (some type of) code-switching typically accounted for approximately 4%–30% of the variance in their performance on inhibitory control tasks (see Hofweber et al., 2019, 2020a,b; López-Penadés et al., 2020). A *post-hoc* power analysis for multiple linear regressions (G*Power version 3.1; Heinrich Heine University Düsseldorf, Germany) was thus computed. Assuming a medium effect size of *f^2^* = 0.15 (roughly equivalent to approximately 13% variance explained) and accounting for the total number of predictors included in the regression analyses (*n* = 10; see section 3.3.4), statistical power of 99% is expected for our sample size of *n* = 225.

#### 3.1.1. The Canadian dual-language interactional context

Recall that we presuppose that the participants in the current study originated from an environment that is highly conducive to the dual-language interactional context. These bilinguals must often operate in a coupled control mode (Green and Wei, 2014), and thus, are more likely to have trained the substrates which underlie the executive functioning system. Green and Abutalebi (2013) describe the dual-language interactional context by stating that individuals who exist in these types of environments typically use different languages when they interact with different co-interlocutors; speakers may switch between their languages within a single conversation, but seldom do so within a single sentence (recall Marthe’s profile in (1b), section 1.2). Code-switching researchers maintain that language alternations of this type (i.e., inter-sentential but not intra-sentential switches) are “particularly frequent in stable bilingual communities with a tradition of language separation” (Muysken, 2000, p.8).

This description accurately matches the situation of Francophones in Canada, particularly in the province of Ontario. While Francophones have been living in the region for centuries (see Allaire, 2007), a history of social, political, and economical tensions has strained the language groups (Darroch and Ornstein, 1980; Jean-Pierre, 2018). Canada implemented the Official Languages Act in 1969 (see Martel, 2019), a statute declaring that both English and French were equal official languages of the country. Nevertheless, Canada’s turbulent cultural-linguistic history leaves its scars on many bilingual communities today, wherein language separation continues to be the norm, particularly in the public sphere. For instance, an amalgamation of several Canadian censuses reveals that L1-French Ontarians used “French only” or “mainly French” approximately 20% of the time at work and in the community (Statistics Canada, 2010). Recent studies also indicate that Francophones face linguicism in Ontario (where the majority language is English) and Anglophones face linguicism in Québec (where the majority language is French; see Jean-Pierre, 2018, pp.519–520 for testimonies).

These types of attitudes have likely contributed to the stigmatization of French-English code-switching. Indeed, while some bilingual communities take pride in their code-switching habits (e.g., Poplack, 1988), mixing languages in the Canadian context is often devalued. As can be seen in (2), Internet users opine that Frenglish/franglais is used as a ‘last-resort’ strategy, by speakers of English with poor French competency (2a, c), or speakers of French with poor English competency (2d); it is rarely attributed to high or balanced bilingual fluency. Furthermore, the general public also tends to believe that code-switching is lazy or careless (2b, c, e).

(2) **Bilinguals’ feelings about Frenglish/*franglais*** (all examples obtained from online forums; emphasis added).
  - “[Franglais is] usually employed in the presence of *lower-level French students* or when one cannot think of the French for a word or phrase.”
  - “[Franglais is] used most commonly by Canadian high school students […] in order to suit (a) the *speaker’s knowledge* of the language; (b) the *speaker’s laziness in regard to full translation* or (c) the speaker’s *desire to piss off their French/English teacher*.”
  - “[Franglais is spoken by] an Anglophone or native English speaker who *speaks French as a second language* [because they are]: (a) *too lazy to think of the correct translation* for what they are saying, and therefore incorporate English words into their sentences, or (b) make a direct word-for-word translation of what they wish to say, in which *case the translated term does not make sense* in French. A common thing done by *French Immersion students*….”
  - “[Frenglish is] used by the youngsters or by French folks *who cannot speak English very well*.”
  - “Is [franglais] a *collective mental laziness* or simply a *lack of interest in speaking our language well*? And what about Anglicisms? I have the impression that many think that by adding bits of English it makes them look intelligent *when it actually shows their ignorance of their own language*. […] How sad!” (translated from French).

Altogether, it appears that the Francophone Canadian environment wholly fits the blueprint for a dual-language interactional context. French Canadians form a stable bilingual community, but the region’s history reinforces the tradition of language separation, particularly at the community-level. This does not mean that individuals from this environment do not code-switch (see for instance, Gosselin and Sabourin, 2021); rather, it implies that when they do mix between their languages, the phenomenon is likely deliberate or circumstantial. Jean-Pierre (2018) dubs this population as “linguistic straddlers:” Canadian Francophones “straddle” their two languages, and may choose one over the other “as a strategy to deflate tense linguistic situations or to avoid linguistic stigmatization altogether” (p. 516; see also Magnan, 2012). This type of process doubtlessly necessitates cognitive control, the topic of the current study.

#### 3.1.2. Demographic and general language background of the participant sample

The participants from the current study completed the online adaptation of an extensive demographic and language background questionnaire developed within the Canadian French context (Sabourin et al., 2016; available in the Gorilla Open Materials). As can be seen in Table 1, the participants ranged in educational levels. The questionnaire also asked the participant about their experience with video games, as previous research suggests that frequently playing video games may lead to enhanced cognitive control (Bialystok, 2006). However, in our sample, experience with video games was rather low. While more than half self-reported French as a singular L1, the participants’ current most dominant language was close to equally divided between French and English. The large majority of the sample reported currently residing in Ontario.

**Table 1:** Participant demographic background. Percentages indicated with n in parentheses.

	1	2	3	4	5
Highest level of education obtained^a^	36.9% (83)	42.2% (95)	18.2% (41)	1.8% (4)	0.9% (2)
Video-game habits^b^	36.4% (82)	48.0% (108)	3.6% (8)	9.8% (22)	2.2% (5)
Current province of residence^c^	Ontario	Québec		Other	
	82.2% (185)	15.6% (35)		1.8% (4)	
Self-reported L1	Only French	Only English		Both	
	54.2% (122)	19.6% (44)		26.2% (59)	
Self-reported dominant language	52.0% (117)	48.0% (108)			

a Education: 1 = high school; 2 = 1–2 years post-secondary; 3 = 3–4 years post-secondary; 4 = master’s; 5 = doctorate.

b Video-games: 1 = never; 2 = once in a while; 3 = at least once a week; 4 = almost every day; 5 = every day.

c One data point (0.4%) is missing.

The participants’ exposure, proficiency, and usage of both French and English are presented in Table 2. Observably, the participants generally acquired French earlier than English (Cohen’s *d* = 0.37) and were exposed to more French during infancy (*d* = 0.33). However, they reported currently using English substantially more often than French (*d* = 0.31). Overall, the sample was equally as proficient in English and French (*d* = 0.02). This language profile is quite representative of the Franco-Ontarian reality. Indeed, Francophones in Ontario are a minority group (see Statistics Canada, 2010) and tend to be exposed to French primarily through their family and schooling. As they age, Franco-Ontarians are likely to be in high contact with the majority language of the province, English. For instance, data from a series of Canadian censuses indicate that, among Ontarians who declare that French is their first official language spoken, only 49.5% reported that French was their “main language” at the time of the census (Statistics Canada, 2010). This same sort of ‘reversal’ in language habits was exhibited by the participant sample in the current study.

**Table 2:** Participant language background.

	*M* for French (*SD*)	*M* for English (*SD*)	*t*-value (*p*-value)
Age of first exposure in years	0.76 (1.65)	2.14 (2.87)	5.49 (<0.001)
Percent exposure during infancy^a^	63.3 (36.0)	40.3 (36.5)	4.89 (<0.001)
Overall current proficiency^b^	4.59 (0.61)	4.57 (0.58)	0.35 (0.73)
Oral comprehension	4.77 (0.52)	4.78 (0.48)	0.30 (0.76)
Oral production	4.52 (0.77)	4.52 (0.72)	0.06 (0.95)
Writing	4.39 (0.83)	4.49 (0.73)	1.35 (0.18)
Reading	4.70 (0.60)	4.66 (0.62)	0.65 (0.52)
Pronunciation	4.57 (0.78)	4.39 (0.86)	2.25 (0.03)
Current daily use (%)	41.8 (26.1)	57.9 (26.0)	4.62 (<0.001)

a Infancy is described as the time period between 0 and 24 months.

b Proficiencies are self-reported based on the following scale: 0 = very low, 1 = low, 2 = intermediate, 3 = advanced, 4 = near-native, 5 = native.

Note that a portion of participants reported at least some knowledge of a third (35.6%) or fourth (5.8%) language. These participants were deemed to fit the inclusion criteria of the study, since they possessed lower than advanced proficiency in their additional languages (L3: *M* = 1.0 out of 5, *SD* = 0.8; L4: *M =* 0.9 out of 5, *SD* = 0.7) and reported virtually no current daily usage of their L3s and/or L4s (*M* = 0.7%, *SD* = 2.5%). As such, the sample from the current study will be referred to as bilinguals.

#### 3.1.3. Code-switching habits of the participant sample

**Figure 1:**
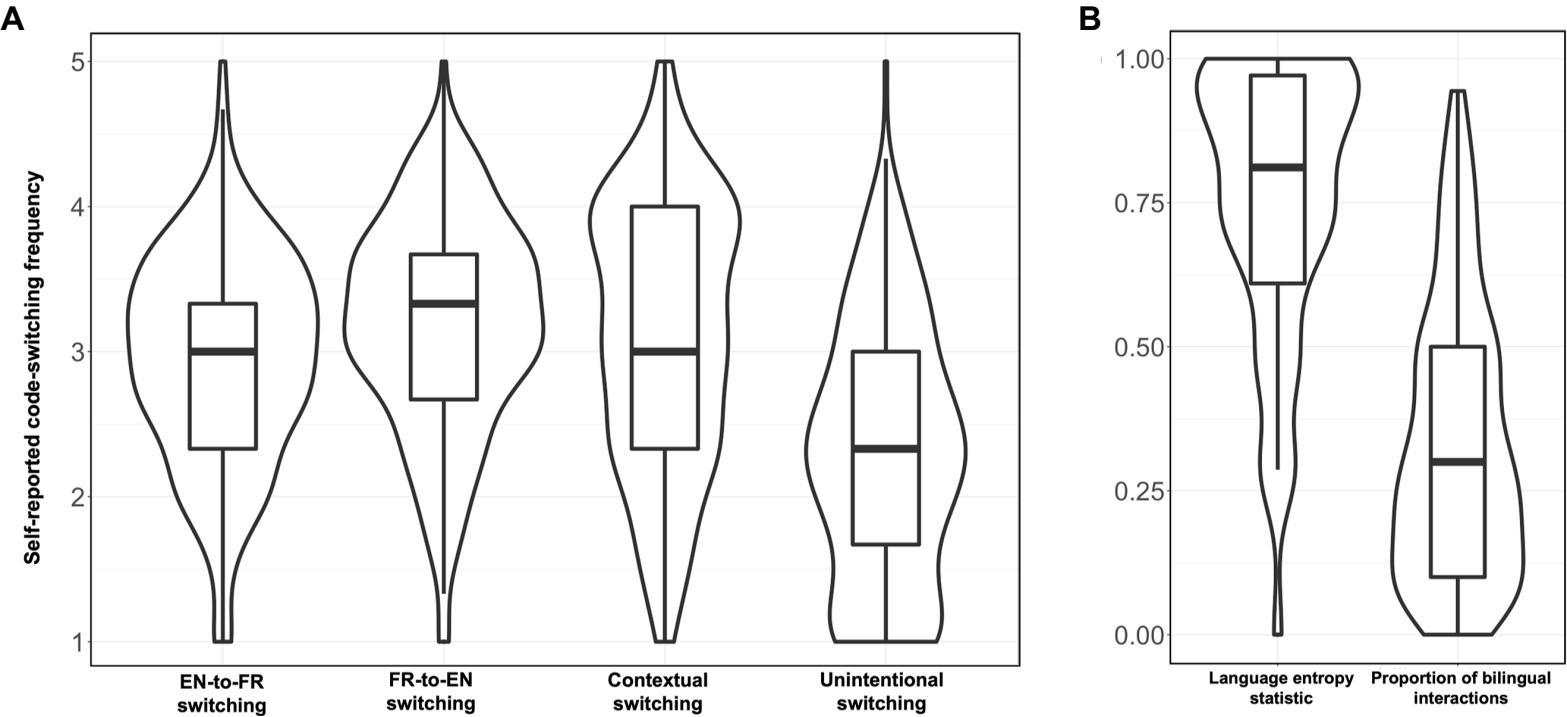
**(A)** Individual variation in code-switching habits (BSwQ components; Rodriguez-Fornells et al., 2012) for all participants. **(B)** Individual variation in the language entropy statistic and proportion of bilingual interactions for all participants. An entropy value of 0 signifies complete linguistic compartmentalization; 1 indicates complete linguistic integration (Gullifer and Titone, 2020). For each variable, the thick black line represents the median, the outline of the box represents the inter-quartile range, the whiskers of the boxplot represent the maximum and minimum values, and the violin outline illustrates the density of the data.

Participants also completed questionnaires assessing their code-switching habits. In particular, the participants completed an online French-English adaptation of the Bilingual Switching Questionnaire (BSwQ: Rodriguez-Fornells et al., 2012). The BSwQ computes four code-switching components by averaging the responses to three questions for each component. English-to-French and French-to-English code-switching components reveal how often participants tend to switch in a given direction (e.g., “*When I cannot recall a word in English/French, I tend to immediately produce it in French/English*.”). For instance, in minority-language contexts, it is typical to observe more switches toward the majority language than vice versa (Lantto, 2015). Contextual switching “assesses the frequency of switches in particular situations” (e.g., “*There are situations in which I always switch between the two languages*.”); unintended switching measures “the lack of awareness of language switches,” when a switch occurs outside of sociolinguistic motivations (e.g., “*It is difficult for me to control the language switches I introduce during a conversation;*” Rodriguez-Fornells et al., 2012: 4). The responses to the questionnaire are presented in Table 3 and Figure 1. Though we did not frame our research questions or hypotheses around language dominance, preliminary analyses revealed important differences in switching habits according to this factor. For this reason, separate data for these groups is presented in Table 3 and dominance was included as a predictor in our statistical analyses (see section 3.3.4).

**Table 3:** Participants’ code-switching habits. Means with standard deviations in parentheses.

		Collapsed (*n* = 225)	FR-dominant (*n* = 117)	EN-dominant (*n* = 108)
Bilingual interactions (%)		33.5 (23.6)	40.2 (23.7)	26.2 (21.4)
BSwQ components	English-to-French (/5)	2.95 (0.73)	3.19 (0.64)	2.69 (0.75)
	French-to-English (/5)	3.18 (0.74)	2.93 (0.75)	3.45 (0.63)
	Contextual switch (/5)	3.10 (0.92)	3.24 (0.91)	2.96 (0.90)
	Unintended switch (/5)	2.30 (0.88)	2.50 (0.87)	2.09 (0.84)

BSwQ component values are based on self-reports using a 5-point Likert scale (1 = “never,” 5 = “always”).

A 2 × 2 ANOVA (including the factors of Switching Direction: English-to-French, French-to-English; and current Dominant Language: French, English) revealed that the participants generally code-switch significantly more often into English than into French [main effect of Direction: *F*(1,446) = 14.544, *p* = 0.0002, *η^2^* = 0.028], though this effect was modulated by their self-reported current dominant language [Direction*Dominance interaction: *F*(1,446) = 62.739, *p* < 0.0001, *η^2^* = 0.120]. In particular, *post-hoc* comparisons indicated that French-dominant bilinguals switch into French more frequently (*t* = 2.964, *p_holm_* = 0.010), while English-dominant bilinguals reported more French-to-English switches (*t* = 8.136, *p_holm_* < 0.0001).

French-dominant and English-dominant bilinguals did not differ in terms of overall switching rate [no main effect of Dominance: *F*(1,446) = 0.013, *p* = 0.908, *η^2^* < 0.0001]. However, French-dominant individuals noted partaking in more bilingual interactions [*F*(1,223) = 21.286, *p* < 0.0001, *η^2^* = 0.087] and, relatedly, reported more regular contextual switching [*F*(1,223) = 5.450, *p* = 0.020, *η^2^* = 0.024], and unintentional switching [*F*(1,223) = 7.079, *p* = 0.008, *η^2^* = 0.031]. Once again, this language profile appears to reflect the Franco-Ontarian reality. English-dominant individuals are less likely to engage in bilingual interactions as the prevalent language used in society is English. Contrastively, French-dominant bilinguals presumably encounter bilingual contexts regularly, as they are shifting between their dominant language and the majority language of the environment.

Finally, to support our conjecture that the participant sample from the current study originated from a primarily dual-language interactional context, we used the participants’ self-reported current use of French and English (see final row of Table 2) to compute language entropy statistics (see Gullifer and Titone, 2020). Language entropy constitutes a new and innovative way to assess language diversity in a given environment. In a hypothetical bilingual environment, an entropy value of zero reveals that individuals keep their languages completely compartmentalized (similar to a single-language interactional context); an entropy value of 1 suggests that bilinguals integrate their language in every type of interaction (similar to a dense code-switching context; Gullifer and Titone, 2020).

The average entropy statistic for the participant sample in the current study was 0.76 (*SD* = 0.24, range = 0–1). This value is consistent with the dual-language interactional context: though the bilinguals use both of their languages in their day-to-day lives (i.e., unlike what is expected in a single-language context), they do not wholly integrate their languages (i.e., unlike what is expected in a dense code-switching context). Nevertheless, a range of entropy values were recorded among the participants (see Figure 1); for this reason, language entropy was included as a predictor in our statistical analyses (see section 3.3.4).

### 3.2. Procedure and task design

The experiment was created and hosted on the Gorilla Experiment Builder website (Anwyl-Irvine et al., 2020). The participants gave informed consent and completed the background questionnaires in their language of choice (French or English) before proceeding to the experimental tasks. In addition to the two inhibitory tasks included in the present study (Flanker and Stroop), the participants also completed two cognitive flexibility tasks (shifting task and lexical decision task; Gosselin and Sabourin, 2021). All four experimental tasks were accomplished online, in counterbalanced order. A break screen appeared between each task and participants could progress through the experiment at their own pace; there was no set time limit to complete the study. At the conclusion of the study, the participants had the option to give their thoughts or feedback about the testing procedure. Note that the full experimental flow reported in the present study is available for preview and cloning on Gorilla Open Materials: https://app.gorilla.sc/openmaterials/429412. This study was not pre-registered.

#### 3.2.1. Flanker task

The Flanker task (Eriksen and Eriksen, 1974) is widely used to assess non-linguistic inhibitory control skills. We utilized an adapted version of the original Eriksen Flanker task (which we derived from a Gorilla sample: see footnote 1) wherein a central arrow is flanked by four horizontally-aligned symbols (two on each side); the participant is asked to identify the direction (left or right) of the center arrow. In congruent trials, flanker arrows face the same direction as the central target (left: 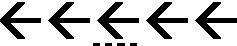, right: 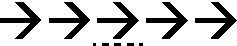). In incongruent trials, flanker arrows face the opposite direction of the target (left: 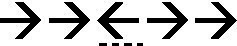, right: 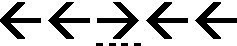). Finally, in neutral trials, the central target is flanked by simple dashes (left: 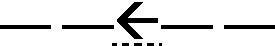, right: 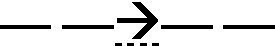). Incongruent trials are expected to generate longer RTs, as the participant must ignore conflicting information. In contrast, congruent trials should be indexed by the shortest RTs, as faciliatory or conforming information is presented alongside the target.

The current study included 100 congruent, 100 incongruent, and 100 neutral trials (split evenly among left-facing and right-facing targets). There were 10 practice trials at the beginning of the task. To keep the experiment interactive, participants saw their ‘score’ (proportion of correct responses) at the end of the practice trials and at the end of the task. Participants were given the option to take a break every 50 trials. Three additional trials, excluded from the data analysis, followed each break; this design was implemented in order to avoid excessive loss of data. In total, each participant saw 300 experimental targets, fully randomized for each participant.

**Figure 2:**
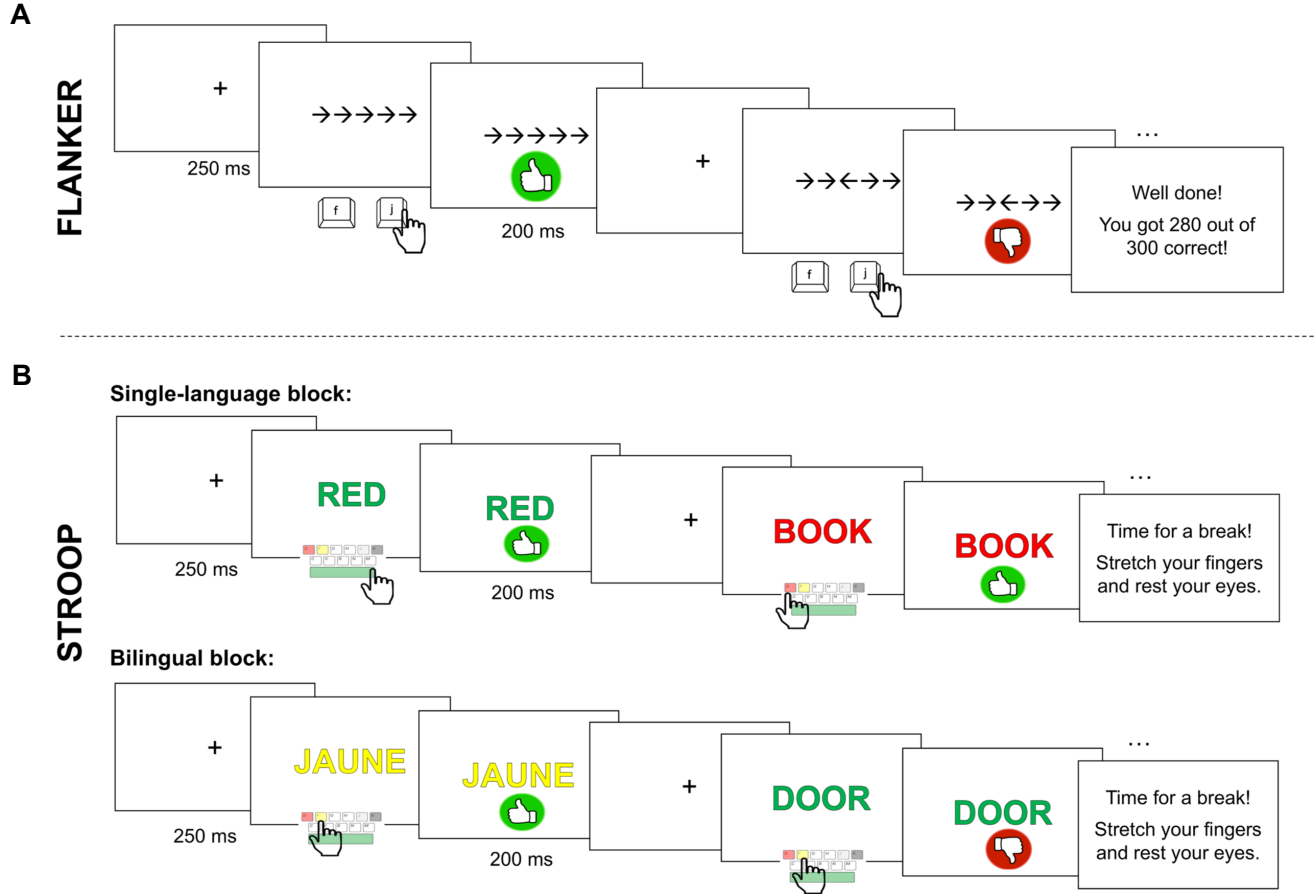
Procedure for the Flanker **(A)** and Stroop **(B)** tasks. Both the single-language and bilingual blocks are depicted for the Stroop task.

A single trial is depicted in the top panel of Figure 2. A fixation cross was first presented for 250 ms. Next, the target and its flankers appeared in black font over a white screen. To reduce cognitive load, participants were instructed to press “f” (the left-most keyboard option) with their left hand when the target-arrow faced left, and to press “j” (the right-most keyboard option) with their right hand when the target faced right. The task flow did not proceed until a keyboard response was selected. Participants received feedback for 200 ms after each individual trial. A green thumbs-up appeared in the case of correct responses, and a red thumbs-down appeared in the case of an incorrect response. The task took approximately 5–7 min to complete.

#### 3.2.2. Stroop task

The Stroop task (Stroop, 1935) is a task used to measure language-related inhibitory control. During this task, participants see chroma-related words (e.g., red, blue, green) and non-chroma words (e.g., house, door, and book) in different colored fonts. They are asked to identify the color of the font and ignore the meaning of the text. This task can be difficult since reading text is an automatic behavior, while responding according to the font-color is more deliberate and conscious. Similar to the Flanker task, the Stroop paradigm is composed of three critical trial types. In congruent trials, the color of the font conforms to the meaning of the word (i.e., “RED” written in red); these trials should generate the quickest response times, as the reflexive reading behavior facilitates the answer for the target. In incongruent trials, the color of the font conflicts with the meaning of the word (i.e., “RED” written in green). Longer response times are expected, since participants must inhibit the automatic reading behavior in order to answer correctly. Finally, neutral trials are baseline or control items, wherein a non-chroma word is presented in a color-font (i.e., ‘BOOK’ written in red) and thus, both facilitation and inhibition are presumably absent.

The current study utilized a French-English bilingual Stroop task (see Sabourin and Vīnerte, 2015, 2020); the programming was derived from an available Stroop sample on Gorilla.^1^ The chroma words (BLACK/NOIR, GREEN/VERT, RED/ROUGE, WHITE/BLANC, and YELLOW/JAUNE) and non-chroma words (BOOK/LIVRE, DOOR/PORTE, HOUSE/MAISON, SNOW/NEIGE, TREE/ARBRE) were non-cognates in these languages. The task was composed of three blocks. The first two blocks were single-language blocks (i.e., only English stimuli, or only French stimuli), each including 25 congruent trials, 25 incongruent trials, and 25 neutral trials (150 single-language trials total). The order of the single-language blocks was counterbalanced across participants. The final (fixed) block was bilingual: it was composed of 50 congruent trials, 50 incongruent trials, and 50 neutral trials, with each condition split evenly across languages (i.e., half of targets were French items and half were English items).

The experiment began with 25 practice trials. To keep the experiment interactive, participants saw their ‘score’ (proportion of correct responses) at the end of the practice trials and at the end of the task. Participants were given the option to take a break every 100 trials. Five additional trials which were excluded from the data analysis followed each break. In total, each participant saw 300 experimental targets (50 each of congruent/incongruent/neutral English items, and 50 each of congruent/incongruent/neutral French items), randomized within blocks.

A single trial is depicted in the bottom panel of Figure 2. A fixation cross was first presented for 250 ms. Next, the target appeared in uppercase letters (Arial font outlined in black) over a white screen. Participants were instructed to place both hands on the keyboard and to press “d” for a target in red font, “f” for yellow font, “j” for white font, and “k” for black font, or the spacebar for green font. An image of a keyboard depicting these responses remained on the screen during the practice trials; this mnemonic disappeared during the experimental trials. The task was participant-controlled: it did not proceed until a keyboard response was selected. Finally, participants received 200 ms feedback after each individual trial. A green thumbs-up appeared in the case of correct responses, and a red thumbs-down appeared in the case of an incorrect response. The task took approximately 7–10 min to complete.

### 3.3. Data analysis

#### 3.3.1. Data cleaning

We adopted a conservative data cleaning procedure (see Zhou and Krott, 2016). First, trials with incorrect keyboard responses were rejected. Correct trials from the Flanker task were trimmed according to an absolute low cutoff (i.e., “anticipation” cutoff) of 150 ms and an absolute high cutoff (“time out” cutoff) of 1,500 ms (see Hofweber et al., 2020a; Jiao et al., 2020a). For the Stroop task, correct trials with response times below 300 ms and above 2,000 ms were trimmed (see Coderre and van Heuven, 2014 for similar methods). After rejecting data points according to these absolute thresholds, a maximum relative RT value was computed for each participant; RTs above three standard-deviations of each participant’s overall mean were subsequently trimmed (see Jylkkä et al., 2017; Ooi et al., 2018; Hartanto and Yang, 2020; Kałamała et al., 2020; Hofweber et al., 2020b; Jiao et al., 2020b). Finally, participants who did not retain at least 80% of trials for each task after data cleaning were rejected from the final analyses. As such, eight participants were excluded for the Flanker task (remaining *n* = 217); seven separate participants were excluded for the Stroop task (remaining *n* = 218). On average, these data cleaning procedures resulted in the preservation of 94.3% of Flanker trials and 90.6% of Stroop trials [no differences according to Language: *t*(224) = 1.456, *p* = 0.147, *d* = −0.097].

#### 3.3.2. Testing expected condition differences

In inhibitory control tasks, incongruent trials are expected to generate longer RTs than neutral and congruent trials. Congruent trials may also be indexed by more rapid RTs than neutral trials. In order to validate the data, we verified whether these expected differences were observed. Repeated-measures ANOVAs were conducted in JASP (version 0.13, University of Amsterdam) on the RT data of the Flanker and Stroop tasks. Both analyses included the variable of Condition (Congruent, Neutral, or Incongruent). The additional factor of Language of the trial (English or French) was entered into the Stroop analysis. In the case of significant effects, planned comparisons were conducted and corrected for family-wise error rates using the holm adjustment.

#### 3.3.3. Operationalizing inhibitory control

Recall that we are interested in investigating the participants’ executive functioning in relation to their code-switching habits. As is standard in the literature, we operationalize each participants’ inhibitory control abilities by computing the facilitation effect and inhibition effect they display in the Flanker and Stroop tasks. The facilitation effect is quantified by subtracting average RTs to congruent trials from average RTs to neutral trials. High conformity trials (the congruent condition) typically elicit more rapid RTs than neutral trials, so the facilitation effect is expected to be positive. Note that the facilitation effect operationalizes an individual’s ability to monitor goal-relevant info and to skillfully disengage the mechanisms related to inhibition (Linck, 2015); larger facilitation effects (i.e., more positive) reflect higher inhibitory control skills. In contrast, the inhibition effect is quantified by subtracting average RTs to incongruent trials from average RTs to neutral trials. High conflict trials (incongruent condition) typically elicit slower RTs than neutral trials, so the inhibition effect is expected to be negative. The inhibition effect measures an individual’s ability to ignore external lures; a lesser effect (i.e., less negative) indicates better inhibitory executive functioning.^2^

#### 3.3.4. Multiple regressions

To examine the relationship between code-switching habits and executive functioning, we conducted a series of multiple linear regression analyses (see also Soveri et al., 2011; Jylkkä et al., 2017; Paap et al., 2017; Hartanto and Yang, 2019, 2020; Hofweber et al., 2019, 2020a,b; Chan et al., 2020; Kałamała et al., 2020; López-Penadés et al., 2020; Sanchez-Azanza et al., 2020; Treffers-Daller et al., 2020; Jiao et al., 2020b for similar methods). Similar to correlations, multiple regressions examine the relationship between a dependent variable and independent variables of interest. However, multiple regressions possess the substantive advantage of relating multiple independent variables (usually referred to as predictors, covariates, or explanatory variables) to a dependent variable (often called the criterion variable) at once (Plonsky and Ghanbar, 2018). In our case, the regressions were computed on the facilitation and inhibition effects of the experimental tasks. For the linguistic Stroop task, separate regressions were also completed according to the Language of the trial (English or French).

Multiple regressions are particularly suited to the current study, as four separate code-switching components were computed (see Table 3), in line with the standard procedure for the BSwQ; each of these components may act as predictors to the participants’ RT in the experimental tasks. In addition, other demographic and language factors may modulate the bilingual’s RTs, such as their age (Bialystok et al., 2004), their education level (Dorbath et al., 2013), their experience with video games (Bialystok, 2006) and their language-dominance (Gathercole et al., 2014). Several researchers also maintain that a speaker’s language environment (e.g., bilingual interactions, language entropy) may play a role in executive functioning training (e.g., Green and Abutalebi, 2013; Green and Wei, 2014). These predictors may act in tandem with, or independently to, the code-switching components.

As such, the predictors included in the regression analyses were the following: (i) age (16–50), (ii) education level (1 “high school” to 7 “doctorate”), (iii) video-game experience (1 “never” to 5 “every day”), (iv) amount of bilingual interactions (“%biling”: 0.0–1.0), (v) language entropy statistic (0.0–1.0), (vi) language dominance (−4 “very English-dominant” to +4 “very French-dominant”)^3^, (vii) frequency of French-to-English switching (“FR-to-EN”; 0–5), (viii) frequency of English-to-French switching (“EN-to-FR”; 0–5), (ix) contextual switching frequency (“Contextual”; 0–5) and (x) unintentional switching frequency (“Unintended”; 0–5).

A potential issue for multiple regressions is multicollinearity, which occurs when predictors are intercorrelated. Collinearity may affect variable specification in the multiple regression analyses (Derksen and Keselman, 1992; Graham, 2003; Ryan, 2008). That is, if multiple predictors are interrelated, a given predictor may be deemed relevant in the final regression model even though it does not account for the dependent variable. As such, we computed a correlation matrix for our predictor variables and determined that there were no multicollinearity issues (i.e., no correlations between predictors above 0.70; Plonsky and Ghanbar, 2018). Furthermore, the QQ (Quantile vs. Quantile) standardized residuals of each regression was plotted. A visual analysis of these plots confirmed that the RT data was approximately normally distributed, and that it was appropriately described by a linear function. The assumptions of homoscedasticity and of independence of errors (Durbin Watson statistic: 1.775–2.109) were also fulfilled.^4^

Variable specification in the current multiple regression analyses was completed using the stepwise elimination procedure (see also Paap et al., 2017; Hofweber et al., 2019, 2020a,b; López-Penadés et al., 2020). Thus, variable selection began with no predictors (i.e., a null model). In a first “step,” the predictor responsible for the most variance of the dependent variable is checked against the stepping method criteria (a change in R^2^ corresponding to a *p* < 0.05). If the inclusion criterion is not met, the null model remains; if it is satisfied, that predictor is included in the regression. This procedure is then repeated among the remaining predictors (subsequent “steps”): other independent variables can also be included in the equation if they meet the inclusion criterion. Furthermore, at every “step,” the predictors are checked against a removal criterion (a change in R^2^ corresponding to an increase of *p* > 0.10); if this condition is met, that predictor is removed from the equation. As such, the final regression equation is computed when all predictors included in the equation do not satisfy the removal condition, and all predictors not yet included in the equation fail to meet the inclusion condition. We report each step of the model specification which yielded a significant regression equation.

## 4. Results

### 4.1. Expected condition differences and descriptive data

#### 4.1.1. Flanker

**Figure 3:**
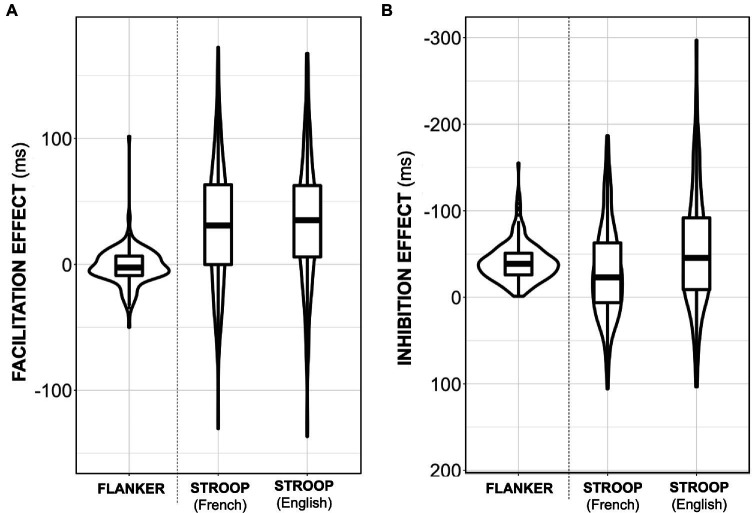
**(A)** Individual variation in the Facilitation effect (neutral—congruent trials) for both tasks. **(B)** Individual variation in the Inhibition effect (neutral—incongruent trials) for both tasks. For each variable, the thick black line represents the median, the outline of the box represents the inter-quartile range, the whiskers of the boxplot represent the maximum and minimum values, and the violin outline illustrates the density of the data. Note that for the inhibition effect, negative is plotted up.

As expected, the condition of the Flanker trial impacted the participants’ RT [main effect of Condition: *F*(2, 432) = 546.105, *p* < 0.0001, *η^2^* = 0.717]. Planned comparisons indicated that incongruent trials elicited significantly longer RTs than congruent trials (*t* = 27.977, *p*_holm_ < 0.0001, *d* = −0.730) and neutral trials (*t* = 29.224, *p*_holm_ < 0.0001, *d* = 0.762). Congruent and neutral trials generated similar RTs (*t* = 1.247, *p*_holm_ < 0.213, *d* = 0.033). This means that, on average, participants failed to exhibit a facilitation effect, but they did display a relatively large overall inhibition effect (see Table 4; Figure 3).

**Table 4:** Descriptive statistics for both the Flanker (*n* = 217) and Stroop (*n =* 218) tasks.

		Congruent	Incongruent	Neutral	Facilitation effect	Inhibition effect
Flanker (ms)		427.80 (49.65)	467.29 (61.70)	426.05 (49.81)	−1.76 (14.63)	−41.18 (22.53)
Stroop	French (ms)	726.37 (103.56)	788.79 (119.68)	760.24 (107.78)	33.86 (55.29)	−28.55 (55.97)
	English (ms)	717.93 (105.36)	804.96 (132.65)	751.96 (105.28)	34.03 (51.84)	−52.99 (68.19)

Means indicated with standard deviations in parentheses.

#### 4.1.2. Stroop

Similar results were observed for the Stroop task [main effect of Condition: *F*(2, 434) = 254.167, *p* < 0.0001, *η^2^* = 0.346]: incongruent trials elicited significantly longer RTs than congruent (*t* = 22.515, *p*_holm_ < 0.0001, *d* = −0.662) and neutral (*t* = 12.286, *p*_holm_ < 0.0001, *d* = 0.361) trials. Unlike in the Flanker task, congruent trials were significantly shorter than neutral trials (*t* = 10.229, *p*_holm_ < 0.0001, *d* = −0.301). Furthermore, an interaction between Condition and Language was observed [*F*(2, 434) = 14.303, *p* < 0.0001, *η^2^* = 0.012]. Simple main effects for Language indicated that incongruent trials elicited much shorter RTs in French than in English (*p* = 0.0008), while congruent and neutral trials were marginally longer in French than in English (congruent: *p* = 0.023; neutral: *p* = 0.037). This means that, on average, participants displayed greater inhibition effects on English trials compared to French trials [*t*(217) = 4.507, *p* < 0.0001, *d* = −0.305], but similar facilitation effects for both languages [*t*(217) = 0.035, *p* = 0.972, *d* = 0.002; see Figure 3]. Nevertheless, both languages displayed the expected differences between the different condition types (see Table 4; Figure 3), validating the study design.

### 4.2. Reliability and convergence

Split-half reliability was measured by separating the trials from each task into two subsets (odd vs. even trials) and computing the average facilitation and inhibition effects of each participant within these individual subsets. An intraclass correlation coefficient (ICC) was then computed between the two subsets for each effect [type ICC(2,1); see Shrout and Fleiss, 1979]. For the Flanker task (*n* = 217), split-half reliability proved to be fair (see Fleiss et al., 2003) for the facilitation effect (ICC = 0.461, 95% CI = 0.296–0.587) and nearly excellent (Fleiss et al., 2003) for the inhibition effect (ICC = 0.745, 95% CI = 0.667–0.805). For the Stroop task (*n* = 218), split-half reliability proved to be poor (Fleiss et al., 2003) for the facilitation effect (ICC = 0.113, 95% CI = -0.159–0.320) and fair for the inhibition effect (ICC = 0.418, 95% CI = 0.240–0.554). Note that individual conditions (i.e., congruent, incongruent, neutral) were found to have excellent split-half reliability in both tasks (ICC_congruent_ > 0.93; ICC_incongruent_ > 0.94; ICC_neutral_ > 0.93).

To quantify the convergence of the EF indices across tasks, Pearson’s correlations were conducted (i.e., Flanker facilitation × Stroop facilitation; Flanker inhibition × Stroop inhibition; *n* = 211). Neither the facilitation effect (*r* = 0.028, *p* = 0.690) nor the inhibition effect (*r* = 0.008, *p* = 0.907) significantly converged across tasks (for similar results see also Paap and Greenberg, 2013; Paap and Sawi, 2014; Jylkkä et al., 2018, 2020; Hartanto and Yang, 2019; Kałamała et al., 2020, etc.). Note, however, that individual conditions (i.e., congruent, incongruent, neutral) were significantly correlated across tasks (congruent: *r* = 0.525, *p* < 0.001; incongruent: *r* = 0.390, *p* < 0.001; neutral: *r* = 0.474, *p* < 0.001). While the lack of convergent validity is criticized in executive functioning research (e.g., Paap and Sawi, 2014), non-convergence may simply reflect the fact that distinct tasks tap into different inhibitory control processes, or different subcomponents of that EF (see also Jylkkä et al., 2020). In our case, perhaps non-convergence is not altogether surprising since we utilized a non-linguistic task and a linguistic task.

### 4.3. Multiple regression

#### 4.3.1. Flanker

**Figure 4:**
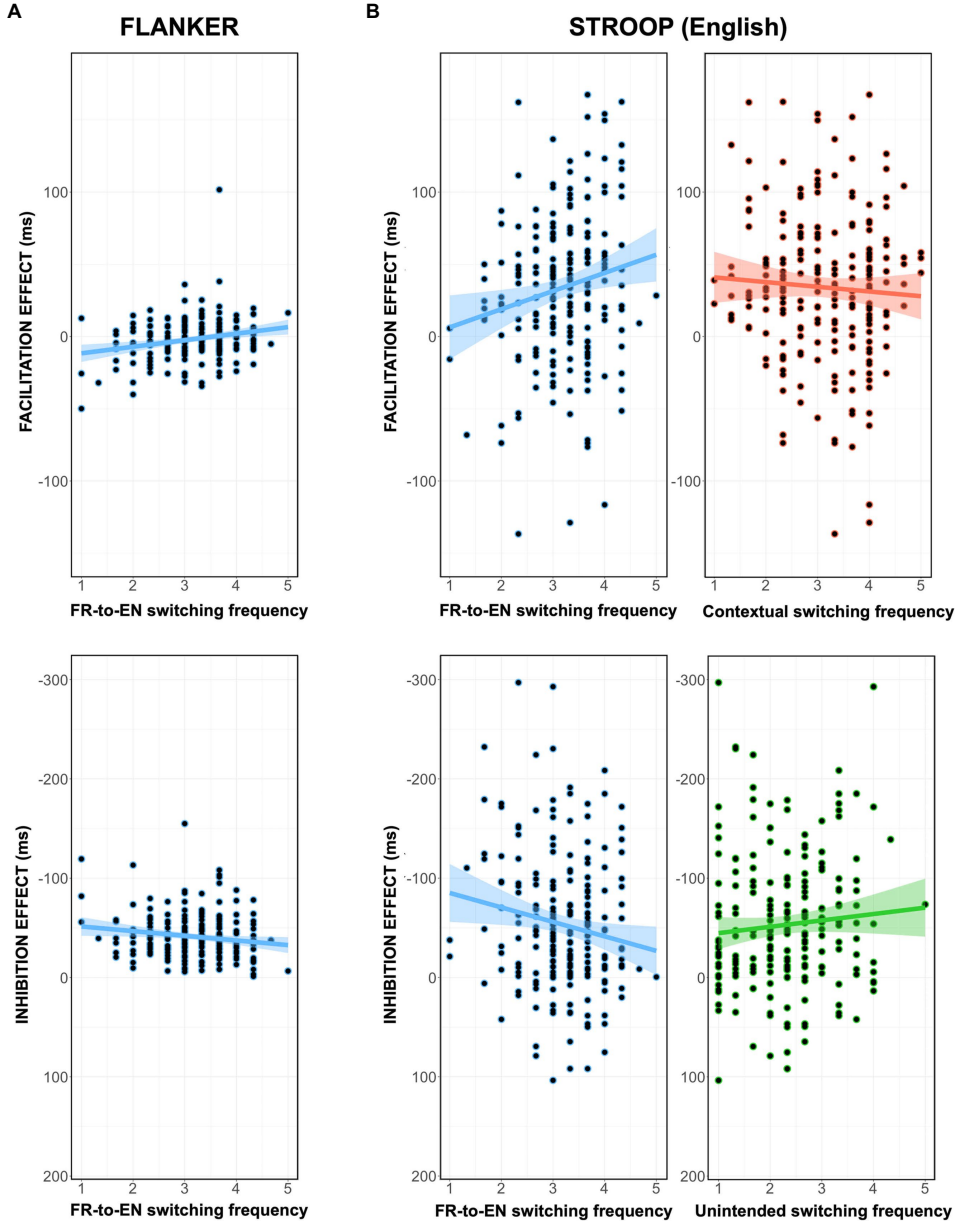
**(A)** Visualization of the significant predictor for the Flanker facilitation effect (top) and inhibition effect (bottom). **(B)** Visualization of the significant predictors for the Stroop facilitation effect (top) and inhibition effect (bottom). The shaded areas surrounding the regression line represent the standard error of the regression. Note that for inhibition effects, negative is plotted up.

The summary of the stepwise regressions for the Flanker task are reported in Table 5A. In the case of the facilitation effect, a significant regression equation was observed for the model containing the predictor of French-to-English code-switching frequency (variance explained: 6.5%). This same independent variable was found to be the most important predictor for the inhibition effect (variance explained: 3.5%). In short, this predictor was found to have a significant positive impact on both dependent variables: bilinguals who reported more frequent French-to-English code-switching exhibited the largest (most positive) facilitation effects and the smallest (least negative) inhibition effects (see Figure 4).

**Table 5:** Summary of the multiple regression statistics for the **(A)** FLANKER task and **(B)** STROOP task.

Dependent variable	Predictor	*R (R^2^)*	*F*	*β* (95% CI)	t
**5A. FLANKER TASK**					
Facilitation	**Step 1**	0.255 (0.065)	14.201^***^		
	FR-to-EN switching			5.164 (2.46–7.87)	3.768^***^
Inhibition	**Step 1**	0.186 (0.035)	7.347^**^		
	FR-to-EN switching			5.755 (1.57–9.94)	2.711^**^
**5B. STROOP TASK**					
Facilitation (French)	None significant	—	—	—	—
Inhibition (French)	**Step 1**	0.150 (0.022)	4.692^*^			Education level			−5.673 (−10.84–−0.51)	2.166^*^
Facilitation (English)	**Step 1**	0.181 (0.033)	6.938^**^		
	FR-to-EN switching			13.360 (3.36–23.36)	2.634^**^
	**Step 2**	0.226 (0.051)	5.483^**^		
	FR-to-EN switching			17.140 (6.52–27.76)	3.183^**^
	Contextual switching			−8.422 (−16.80–−0.04)	1.982^*^
Inhibition (English)	**Step 1**	0.182 (0.033)	7.016^**^		
	FR-to-EN switching			17.543 (4.49–30.60)	2.649^**^
	**Step 2**	0.240 (0.058)	6.240^**^		
	FR-to-EN switching			21.492 (8.14–34.85)	3.173^**^
	Unintentional switching			−12.602 (−23.38–−1.83)	2.306^*^

The following predictors were considered for the stepwise variable specification: (i) age, (ii) education level, (iii) video-game experience, (iv) bilingual interactions, (v) language entropy statistic, (vi) language dominance, (vii) French-to-English switching, (viii) English-to-French switching, (ix) contextual switching, (x) unintentional switching. Absolute *t*-values and unstandardized coefficients (*β*) are reported. ^*^p ≤ 0.05, ^**^p ≤ 0.01, ^***^p ≤ 0.001.

#### 4.3.2. Stroop

The summary of the stepwise regressions for the Stroop task is reported in Table 5B. In French, the multiple regression failed to produce a significant regression equation for the facilitation effect. On the other hand, the inhibition effect (in French) was found to be predicted by the participants’ education level (variance explained: 2.2%). That is, bilinguals who had higher levels of education tended to display larger inhibition effects.

For English trials, both the participants’ facilitation effect (variance explained: 5.1%) and inhibition effect (variance explained: 5.8%) were predicted by the code-switching variables. First, the facilitation effect was positively related to the bilinguals’ propensity to code-switch from French-to-English but negatively associated to their contextual code-switching. Second, the inhibition effect was positively related to the bilinguals’ French-to-English switching frequency, but negatively associated to their propensity to unintentionally code-switch. In short, the predictor of French-to-English code-switching was linked to both larger (more positive) facilitation effects and smaller (less negative) inhibition effects. However, directionally opposite effects concerning contextual switching (facilitation) and unintentional switching (inhibition) appear to mitigate these benefits (see Figure 4).

## 5. Discussion

The presence of so-called “bilingual executive functioning advantages” is still highly contested. The present study opted to lean away from the global, binary perspective of this hypothesis; instead, we examined nuanced bilingual experiences in an effort to determine why some—but not all—bilinguals display inhibitory control benefits. In particular, we focused on the code-switching habits of bilinguals who most frequently operate in a dual-language interactional context. Since this is the environment which is presumed to train a bilingual’s inhibitory control skills (Green and Abutalebi, 2013; Green and Wei, 2014), we hypothesized that more frequent code-switching in this context would lead to inhibitory control advantages. Over 200 French-English bilinguals completed domain-general (Flanker) and language-specific (bilingual Stroop) inhibitory control tasks, as well as a series of extensive demographic and language background questionnaires. While the French trials in the Stroop task yielded no significant results, the findings from both the Flanker and the English trials of the Stroop task converged: participants who self-reported more habitual French-to-English code-switching exhibited inhibitory control advantages (i.e., larger facilitation effects and reduced inhibition effects). However, when it came to language-specific inhibitory control, such benefits appeared to be mitigated by other types of code-switching. These findings will be further discussed in the subsequent sections.

### 5.1. Dual-language switching trains inhibitory control

Results from Flanker and Stroop (English) paradigms demonstrated that switching in a dual-language context may lead to increased goal-monitoring and inhibition abilities (i.e., inhibitory control advantages). These findings are compatible with the idea that (some type of) code-switching positively impacts executive functioning mechanisms, as has been demonstrated in past research (Hofweber et al., 2016, 2019, 2020a; Verreyt et al., 2016; Ooi et al., 2018; Hartanto and Yang, 2020; Jylkkä et al., 2020; Sanchez-Azanza et al., 2020; Kheder and Kaan, 2021).

Recall that the BAH stems from the theory that bilinguals train their EFs when they activate and inhibit their languages. Dual-language code-switchers doubtlessly perform this activation–inhibition shuffle more often than bilinguals who do not code-switch (or bilinguals who opportunistically code-switch, as in a dense-code-switching environment; see section 5.3), and this experience appears to put their EF system into practice. Impressively, this training is not restricted to language-specific activities; inhibitory control advantages are observed even when inhibition and monitoring take place in non-linguistic contexts (i.e., the Flanker task). These findings support previous fMRI research which indicates that the neural substrates involved in language switching overlap with the regions normally associated to domain-general executive functioning (e.g., Abutalebi and Green, 2008); practicing one (i.e., language switching) might thus result in better performance in the other (i.e., executive functioning; see Bialystok et al., 2012).

Note that the bilinguals tested in the present study originated from dual-language interactional contexts, the environment that is hypothesized to train executive functioning mechanisms (Green and Abutalebi, 2013; Green and Wei, 2014). As such, the current findings also support the idea that past null or negative results regarding the impact of code-switching in inhibitory control training (Soveri et al., 2011; Jylkkä et al., 2017, 2018; Ooi et al., 2018; Chan et al., 2020; Kałamała et al., 2020; López-Penadés et al., 2020; Hofweber et al., 2020b) may have been confounded by the previous participant samples’ interactional context of origin. For instance, dense code-switching contexts do not put into practice cognitive mechanisms, as bilinguals can use their language cooperatively and opportunistically (Green and Wei, 2014). Individuals originating from these types of environments likely do not reap the same code-switching-related benefits as do dual-language bilinguals.

### 5.2. The unidirectionality of the code-switching effect

Though multiple code-switching parameters were examined, only the participants’ frequency of *French-to-English* code-switching emerged as a significant positive predictor; this was true of both their domain-general *and* language specific inhibitory control. That is, bilinguals who reported frequently code-switching into English (but not those that reported switching into French) exhibited greater goal-monitoring abilities (i.e., larger facilitation effects) and better inhibition skills (i.e., smaller inhibition effects). Thus, it appears as though code-switching *direction* may play a role in EF training.

Why does habitual French-to-English switching exert a unidirectional influence on the inhibitory control abilities of the participants in the current study? First, it is possible that this asymmetry was observed since French-to-English is simply the code-switching direction that is most likely within the dual-language interactional context that we have described. This is unsurprising, as English is the majority language of the environment, and code-switching tends to occur from the minority language into the majority language (e.g., Lantto, 2015). Indeed, the participants in our sample reported significantly more code-switching into English than into French (see section 3.1.3). In brief, perhaps, French-to-English code-switching frequency consistently emerged as the most important predictor for the participants’ inhibitory control as this is the habit they actually partake in, and is thus the one that provides the most robust EF training.

Second, it is also possible that the unidirectional code-switching effect in the current study is a result of the fact that most of the participants possessed French as an L1. Seminal production research affirms that language switching costs are indeed asymmetrical: in general, researchers believe that an individual’s L1 is more persistent and difficult to suppress (e.g., Meuter and Allport, 1999). This entails that, for bilinguals who are switching between their languages, greater cognitive effort is required to suppress the L1 than to suppress the L2. This phenomenon has also been observed in neuroimaging studies, wherein code-switching in the dominant-to-weak direction elicits electrophysiological processing costs, but the weak-to-dominant direction does not (e.g., Litcofsky and Van Hell, 2017). In the current study, the participants acquired French before English (see section 3.1.2). Thus, perhaps those who reported frequent French-to-English code-switching manifested inhibitory control advantages since they are those who have regular experience with the effortful task of inhibiting their L1 (i.e., in this case, L1-French must be suppressed in order to produce a French-to-English switch). This may also explain why the effects were observed for English trials of the Stroop task, but not for French trials of the Stroop task. Strikingly, the asymmetry persisted even though the participants reported using English to a higher degree than French in their daily lives, and regardless of their computed linguistic dominance.

Overall, while it is possible that frequent code-switching in both directions positively impacts inhibitory control, the observed asymmetry suggests that code-switching likely trains bilingual executive functioning primarily through the frequent suppression of the L1. Perhaps this is unsurprising: if L1-suppression is the most cognitively arduous portion of the code-switching act, it logically produces the most robust cognitive training.

### 5.3. The role of code-switching cognizance in inhibition

Interestingly, the results from the current study also point toward the fact that inhibition training provided by habitual code-switching must involve a certain awareness of this switching behavior. That is, we observed a reduction in language-specific inhibitory control advantages for bilinguals who reported regularly switching between their languages *unintentionally* (e.g., “Without intending to, I sometimes produce the English/French word faster when I am speaking French/English;” I do not realize when I switch the language during a conversation or when I mix the two languages;” “When I switch languages, I [do not] do it consciously;” Rodriguez-Fornells et al., 2012).

In particular, participants who self-reported frequent unintentional switching displayed larger inhibition effects (i.e., reduced inhibition skills) for the English trials (but not French trials) of the Stroop task. Notice that unintentional code-switching mitigated benefits for language-specific inhibitory control, but did not predict the participants’ performance in the domain-general task (though see López-Penadés et al., 2020 for effects on Flanker accuracy). We posit that unintentional switching does not necessarily *reverse* the other types of cognitive training that an individual may obtain. In other words, we believe that unintentional switching is related to smaller inhibitory control advantages since bilinguals who partake in this behavior likely produce less *deliberate* switches, and thus obtain less inhibitory control training. Overall, it appears that code-switching only trains language-specific executive functioning system if it occurs deliberately.

Unintentional switching has, in fact, previously been linked to weak cognitive control, or compared to a type of linguistic “failure” resulting from executive functioning deficits (see Rodriguez-Fornells et al., 2012; Hartanto and Yang, 2020 for discussions). For instance, Festman and Münte (2012) and Festman et al. (2010) separated bilinguals into groups based on their performance in a bilingual picture naming task. Bilinguals with frequent cross-linguistic intrusions (i.e., naming a picture in a non-target language) were labeled as “switchers.” Switchers subsequently displayed lower performance on a series of executive functioning tasks compared to the group of “non-switchers.” The idea is that bilinguals who switch languages ‘accidentally’ (even though they are in a dual-language context, like the sample in the present study) are likely unable to adequately suppress their non-target language.

Importantly, the impact of code-switching cognizance on inhibitory control observed in the current study supports the Adaptive Control Hypothesis: this hypothesis claims that different interactional contexts impose differential cognitive control demands on the bilingual speaker (Green and Abutalebi, 2013). On the one hand, dual-language contexts presumably impose the highest demands, as individuals must monitor their environment and select one of their co-activated but competing languages (Green and Abutalebi, 2013). On the other hand, dense code-switching contexts require little cognitive control, as bilinguals can use their languages cooperatively. Crucially, unintentional code-switching is completely natural in dense code-switching contexts, while the dual-language context assumes an inherent awareness of code-switching behaviors. Thus, our results effectively demonstrate that more dual-language switching leads to increased inhibitory control benefits, but more switching in a dense code-switching context does not.

Finally, we would like to note that unintentional switching behaviors were not related to the participants’ facilitation effect. Rather, participants who reported more contextual code-switching displayed *reduced* goal-monitoring abilities (i.e., smaller facilitation effects). This suggests that the training imbued by the dual-language context is relatively specific. While the dual-language context targets an individual’s ability to actively suppress one language or another, as well as the disengagement of the mechanisms related to inhibition, it is not clear that the deliberate nature of code-switching in the dual-language context trains an individual’s monitoring abilities. Perhaps, bilinguals in a dense code-switching context must still perform goal monitoring as they decide whether to code-switch or not at all. This may explain why the participants’ facilitation effect, unlike the inhibition effect, is not negatively impacted by unintentional switching.

### 5.4. A note of caution: Monolinguals should not be overlooked

Though code-switching habits were found to be significantly related to the bilinguals’ executive functioning abilities in the present study, it is imperative not to overextend the data. The variance explained by the significant predictor(s) in each of the tasks and was approximately between 2–6.0%.^5^ This does not mean that code-switching fails to provide any inhibitory control training, but rather, that switching habits only contribute a moderate piece to a larger puzzle. In other words, we must not assume that code-switching is the be-all and end-all in cognitive benefits; numerous other language and demographic parameters, not investigated here, likely interact with each other as they play a role in bilingual inhibitory control. Future research should continue to collect detailed demographic, social, and language background information about their participant samples in order to disentangle potentially confounding factors and increase the statistical variance explained in bilingual executive functioning studies (see Luk, 2022; Titone and Tiv, 2022 for enlightening discussions).

Moreover, while our findings prompt the conclusion that habitual dual-language code-switching may lead to cognitive advantages, the present study does *not* compare bilinguals to monolinguals. Though switching between languages is a skill that is unique to individuals who speak more than one language, the delineation of what constitutes a “language” can be vague and ambiguous (Haugen, 1966). Perhaps, monolinguals also have the opportunity to participate in linguistic behaviors that may train their executive functioning system, such as switching between dialects, registers, cultural identities, or using borrowings (e.g., Alrwaita et al., 2022; Treffers-Daller et al., 2020; though see Poarch et al., 2019). Thus, the present study does not directly support the bilingual advantage hypothesis as it relates to the juxtaposition of bilinguals vs. monolinguals.

With this in mind, it is of primordial importance to ensure that, as psycholinguists, we are not tracking a lopsided target when it comes to bilingual advantages. That is, as we continue to study bilingualism from more nuanced and experience-oriented perspectives, we must not in the same breath reduce monolinguals to a monolithic set. Monolinguals also experience diversity in their language experience (e.g., Bice and Kroll, 2019), and within-subject studies for this group are well-needed. When it comes to examining language usage, both monolinguals and bilinguals are complex populations that cannot be reduced to static and immutable factions.

### 5.5. Conclusion

The current study examined whether code-switching in a dual-language interactional context may train a bilingual’s inhibitory control. French-English bilinguals completed two inhibitory control tasks. In brief, the findings indicated that frequent dual-language code-switchers experience language-specific and domain-general benefits in goal-monitoring and inhibition, but only unidirectionally (i.e., if they frequently code-switch into the majority language). When it comes to language-specific inhibition skills, code-switching is uniquely beneficial if it is deliberate. By contrast, regular unintentional switching is linked to reduced inhibition benefits. We conclude that the bilinguals’ interactional context is thus of primary importance, as intentional code-switching is conducive to a dual-language context and unintentional code-switching is more typical in a dense code-switching environment. Overall, the results suggest that deliberate code-switching puts into practice the mechanisms underlying inhibitory control, and may thus contribute to bilingual advantages. Habitual, dual-language code-switchers are essentially ‘linguistic athletes’ trained in cognitively advantageous language usage. While this study does not directly compare code-switchers to monolingual participants, it highlights the necessity of adopting a less dichotomized view of the bilingual advantage hypothesis.

## Data availability statement

The datasets presented in this study can be found in online repositories. The names of the repository/repositories and accession number(s) can be found at: https://osf.io/qdsv4/?view_only=66344c9efb354c75a6ff4368a28c27a0 and https://app.gorilla.sc/openmaterials/429412.

## Ethics statement

This study received full ethics approval from the University of Ottawa’s Research Ethics Board (file #S-01-21-6539). The patients/participants provided their written informed consent to participate in this study.

## Author contributions

LG and LS conceptualized and designed the study. LG collected the data, conducted the analyses, and wrote the manuscript. LS supervised these tasks. All authors contributed to the article and approved the submitted version.

## Funding

LG was supported by a Joseph-Armand Bombardier Scholarship from the Social Sciences and Humanities Research Council of Canada as well as an Ontario Graduate Scholarship.

## Conflict of interest

The authors declare that the research was conducted in the absence of any commercial or financial relationships that could be construed as a potential conflict of interest.

## Publisher’s note

All claims expressed in this article are solely those of the authors and do not necessarily represent those of their affiliated organizations, or those of the publisher, the editors and the reviewers. Any product that may be evaluated in this article, or claim that may be made by its manufacturer, is not guaranteed or endorsed by the publisher.

## Footnotes

^1^ Flanker sample: https://app.gorilla.sc/admin/task/3850/editor; Stroop sample: https://app.gorilla.sc/admin/task/16735/editor.

^2^Many (perhaps most) studies instead compute an interference or conflict effect, wherein high conflict trials are subtracted directly from high conformity trials (e.g., Hofweber et al., 2016; Jylkkä et al., 2017, 2018; Hartanto and Yang, 2019; Jylkkä et al., 2020; Sanchez-Azanza et al., 2020). This measure is usually interpreted in the same way as the inhibition effect, but it does not allow for an operationalization of facilitation. As stated by Linck (2015: 2), "this assumes that better inhibitory control abilities will only be reflected in enhanced deployment of inhibition on conflict trials" though "better inhibitory control may also influence performance through more efficient *disengagement* of inhibitory mechanisms, which may manifest on non-conflict trials."

^3^The predictor of dominance was computed by examining each of the following characteristics: self-reported dominant language, age of exposure (AoE), current proficiency, and current daily usage. For each of these characteristics, an asymmetry weighted toward English (e.g., earlier AoE, higher proficiency, or larger proportion of current usage for English) was assigned a value of −1. An asymmetry weighted toward French was assigned a value of +1. If the characteristic was symmetrical in each language (i.e., the same AoE, proficiency or degree of usage for English and French), it was assigned a value of zero. As such, the participant’s dominance ranged on a scale of −4 to +4 (*M* = −0.2, *SD* = 3.1), with −4 representing the most English-dominant bilinguals and + 4 representing the most French-dominant bilinguals. This procedure was adopted in order to avoid high collinearity between each individual language background feature (i.e., AoE, proficiency, degree of usage…).

^4^Note that anonymized data summary files are available on OSF.

^5^This moderate variance explained may also be due to the fact that well-established inhibitory control tasks like Flanker and Stroop tend to possess low between-subject variability (Hedge et al., 2018). In our case, the coefficients of variation (CV) in our data were the following: Flanker facilitation: CV = -8.32; Flanker inhibition: CV = -0.55; Stroop French/English facilitation: CV = 1.63/1.52; Stroop French/English inhibition: −2.00/−1.29. Thus, it is possible that the variance explained is low since there is little inter-subject variance to begin with.

## Abbreviations

BAH: Bilingual advantage hypothesis
CV: Coefficient of variation
EF: Executive function
ICC: Intraclass correlation coefficient
RT: Reaction time
